# Differential gene expression profile of multinodular goiter

**DOI:** 10.1371/journal.pone.0268354

**Published:** 2022-05-20

**Authors:** Wenberger Lanza Daniel de Figueiredo, Eraldo Ferreira Lopes, Deborah Laredo Jezini, Lorena Naciff Marçal, Enedina Nogueira de Assunção, Paulo Rodrigo Ribeiro Rodrigues, Adolfo José da Mota, Diego Monteiro de Carvalho, Spartaco Astolfi Filho, João Bosco Lopes Botelho

**Affiliations:** 1 College of Medicine, Nilton Lins University, Manaus, Amazonas, Brazil; 2 Coari Institute of Health and Biotechnology, Federal University of Amazonas, Coari, Amazonas, Brazil; 3 Department of Internal Medicine, Federal University of Amazonas, Manaus, Amazonas, Brazil; 4 Institute of Biological Sciences, Federal University of Amazonas, Manaus, Amazonas, Brazil; 5 School of Health Sciences, State University of Amazonas, Manaus, Amazonas, Brazil; Brigham and Women’s Hospital, Harvard Medical School, UNITED STATES

## Abstract

**Introduction:**

The goiter, a neglected heterogeneous molecular disease, remains a major indication for thyroidectomies in its endemic regions.

**Objectives:**

This study analyzed differential gene expression in surgical specimens diagnosed with multi nodular and compared the data to that of thyroid tissue without multinodular goiter from patients undergoing thyroidectomy in Manaus-AM, Brazil using RNA-seq technology.

**Methodology:**

The transcriptome information of the surgical specimen fragments with and without multinodular goiter was accessed by Illumina HiSeq 2000 New Generation Sequencing (NGS) using the RNA-seq NEBNext^®^ Ultra^™^ RNA Library Prep Kit for Illumina^®^—#E7530L protocol and differential gene expression analysis.

**Results:**

Differences were found between the gene expression profiles of the diseased tissues and those of the healthy control tissues; at least 70 genes were differentially expressed. The *HOTS* gene was expressed only in multinodular goiter tissues (*p* < 0.05).

**Conclusion:**

These results demonstrate that the gene expression profile of multinodular goiter is pro-tumoral and that *HOTS* can play a central role in multinodular goiter development.

## Introduction

Partial or total removal of the thyroid gland affected by goiter is one of the most commonly performed surgeries in medical practice. The role of goiter as a risk factor for well-differentiated thyroid carcinoma is unclear; however, the prevalence of incidental carcinoma in patients operated for goiter in endemic areas is 10–12% [1, 2], which is greater than the overall prevalence of the disease (5.1%) [3].

Although goiter is the main indication for thyroidectomy in goitrogenic geographic areas, its molecular-genetic component has been scarcely studied compared with that of thyroid carcinoma. In addition, studies conducting massive sequencing for thyroid nodular diseases have focused on well-differentiated and undifferentiated thyroid carcinoma [4, 5].

Transcriptome analysis, which is gaining prevalence in studies on tumor diseases, allows a better understanding of gene expression profiles in tissues under different conditions, including the knowledge of non-coding RNAs (ncRNAs), monoallelic expression of imprinted genes, and several transcriptional phenomena, such as fluctuations in the expression of non-constitutive sequences [6].

The performance of ncRNAs, such as the products of the H19 gene, and their relationship with several types of cancer are well reported in the literature. This gene, which is never expressed beyond the embryonic period in normal conditions, has high expression in tumors related to tissue hypoxia and cancer. Aberrant expression patterns of this sequence occur in breast cancer [7] and melanoma [8].

In lung neoplasms, high expression of H19 is related to the epithelial-mesenchymal transition [9]. Its action on metabolic and cell cycle pathways is thought to be involved in the modulation of a pro-tumor state [10–12].

The need for a preoperative diagnosis due to gaps in the Bethesda cytological classification from fine needle aspiration (FNA) of thyroid nodules and the advent of molecular studies of these diseases have allowed the development of molecular tests for well-differentiated thyroid carcinoma, notably based on the identification of *BRAF* and *RAS* mutations as well as *RET/PTC* and *PAX8/PPARy* rearrangements [13, 14], among others such as Afirma GEC^®^, ThyGenX TEST^®^, and ThyroSeq TEST^®^, all without relevant application for multinodular goiter.

This unprecedented study presents the occurrence of differentially expressed genes between tissues affected by multinodular goiter and disease-free tissues (hereafter referred to as controls) from specimens collected in a geographical area (Amazonas, Brazil) endemic for the disease.

## Method

This study was approved by the Human Research Ethics Committee of the Adriano Jorge Hospital Foundation under CAAE 16463813.9.0000.0007 on June 1, 2013.

The study included transcriptome sequencing of two thyroid tissue fragments with multinodular goiter and one control tissue fragment from patients operated in a multinodular goiter endemic region (Manaus, AM, Brazil).

The thyroid fragments used in this study each measured 1 cm^3^ and were obtained directly from the surgical specimen after its removal from the cervical region by thyroidectomy. Tissue in the control group was obtained from thyroid tissue fragments from patients with thyroid adenoma, from a region of the thyroid gland 1.5 cm away from the nodule. Tissues were confirmed disease-free by pathology service analysis afterwards.

Immediately after collection, the fragments were stored in microcentrifuge tubes containing the preservative RNAlater^™^ Stabilization Solution (Thermo Fisher) in a -80 °C deep freezer until histological classification of the specimen by a pathologist as disease-free tissue or tissue with multinodular goiter.

The total RNA was prepared with TRIzol^®^ Reagent (lifetechnologies^™^) protocol, following the manufacturer’s recommendations. All following steps to transcriptome sequencing was performed by GenOne Soluções em Biointecnologia Facility (Rio de Janeiro, Brazil). RNA libraries were validated in an Agilent 2100 Bioanalyzer using the RNA 6000 nano Assay. The cDNA libraries were constructed by using NEBNext^®^ Ultra^™^ RNA Library Prep Kit for Illumina^®^—#E7530L RNA-seq protocol with an expected output of 20 GB of data per sample and sequenced in the Illumina HiSeq 2000 platform.

### Data analysis

The sequences exploratory analysis were carried out by the Bioinformatics group from the Central Laboratory of High Performance Technologies in Life Sciences (LaCTAD), State University of Campinas (UNICAMP, SP, Brazil). SRA data accession number: PRJNA810866.

Reference genome mapping (*Homo sapiens* HG38) was performed using Bowtie2 [15], transcript quantification was performed using RSEM [16], and differentially expressed genes were analyzed using DESeq [17].

Differentially expressed genes were analyzed for biological function and protein class using the *Panther* tool [18] available on the *Gene Ontology Consortium* platform (http://www.geneontology.org/) [19], followed by protein-protein interaction analysis with the GeneMANIA tool (http://www.genemania.org/) [20–22].

## Results

**Fig 1:**
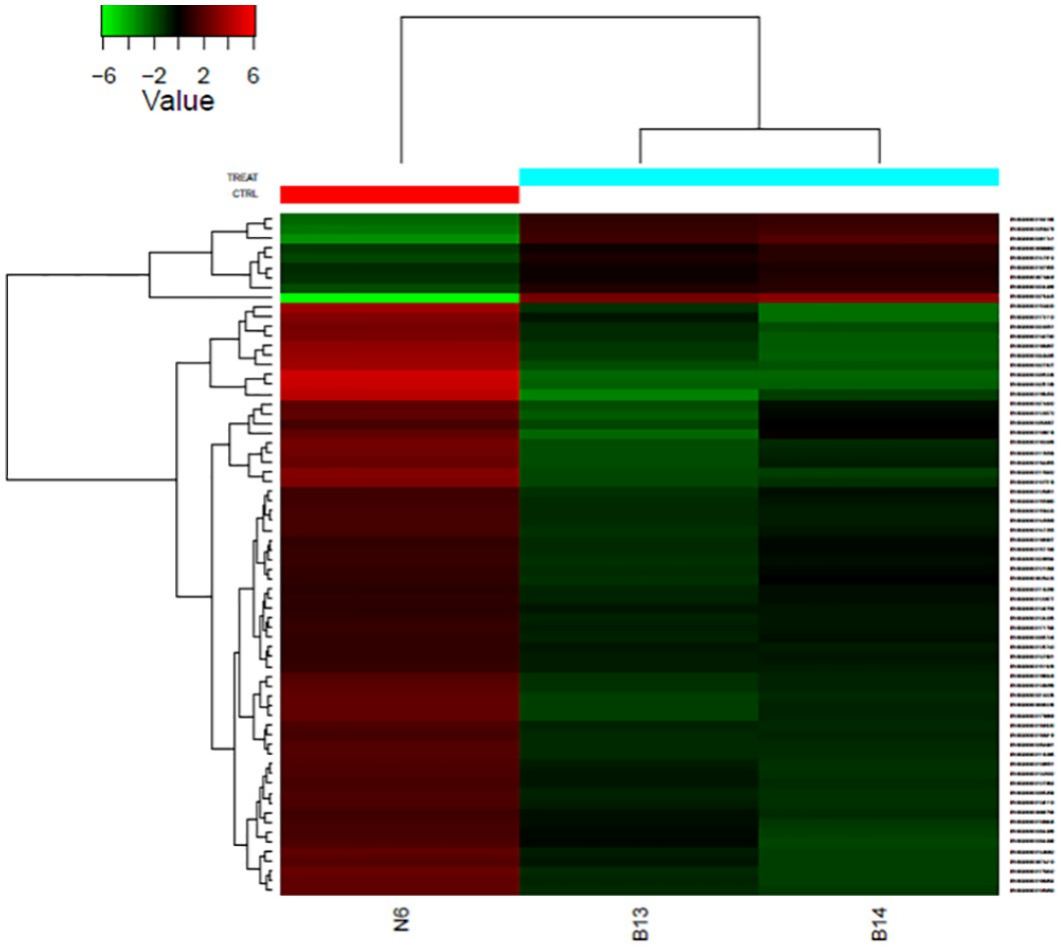
Heat map graph comparing the fold values of the 70 differentially expressed sequences in each sample. Values above the normalized reference value (Z score) are shown in green, the reference value is shown in red, and values close to the reference are represented by a dark tone. Each column represents a sample, and each row represents a sequence. N6, control tissue; B13 and B14, goiter tissues.

The differential expression analysis of two tissue fragments with multinodular goiter and a control tissue identified 65 differentially expressed genes and five pseudogenes, of which 61 were down-regulated and nine up-regulated in thyroid tissue with multinodular goiter compared with the control tissue (Fig 1).

The 70 differentially expressed gene sequences were classified into 62 protein-coding genes; five pseudogenes (*SORD2P*, *PI4KAP1*, *ZBTB45P1*, *TMSB4XP4*, and *PKD1P5*); and three sequences related to the pre-mRNA of the transcription factor *PRPF31* (*RP11-1212*^*a*^*22*.*1*, *RP11-514P8*.*6*, and *RP11-958N24*.*2*), indicating that one sequence was a product of the imprinted H19 locus encoding the HOTS nucleolar protein [Table 1].

**Table 1:** List of genes differentially expressed in multinodular goiter and disease-free tissue when *p* < 0.05 (5%).

Gene symbol	Gene description	FC	Interpretation
HOTS	H19 opposite tumor suppressor	Inf*	*Up-regulated*
SORD2P	Pseudogene	4.18	*Up-regulated*
C4B	Complement C4-B	2.86	*Up-regulated*
C2CD4C	C2 calcium-dependent domain-containing protein 4C	3.86	*Up-regulated*
C241377.2	Protein LOC100996720	5.71	*up-regulated*
CPXM1	Probable carboxypeptidase X1	2.11	*up-regulated*
NAPRT	Nicotinate phosphoribosyltransferase	2.37	*up-regulated*
ST6GAL1	Beta-galactoside alpha-2,6-sialyltransferase 1	1.86	*up-regulated*
COL14A1	Collagen alpha-1(XIV) chain	1.62	*up-regulated*
HSPA6	Heat shock 70 kDa protein 6	-4.21	*down-regulated*
C1QL4	Complement C1q-like protein 4	-5.05	*down-regulated*
PLCD4	Phospholipase C	-3.82	*down-regulated*
ERRFI1	ERBB receptor feedback inhibitor 1	-2.84	*down-regulated*
PCP4L1	Purkinje cell protein 4-like protein 1	-5.27	*down-regulated*
ATRNL1	Attractin-like protein 1	-4.34	*down-regulated*
MT1H	Metallothionein-1H	-2.71	*down-regulated*
ABCA13	ATP-binding cassette sub-family A member 13	-3.32	*down-regulated*
DNAJB1	DnaJ homolog subfamily B member 1	-2.46	*down-regulated*
CA12	Carbonic anhydrase 12	-2.89	*down-regulated*
PKD1P5	Pseudogene	-2.89	*down-regulated*
RP11-958N24.2	Uncharacterized	-5.83	*down-regulated*
ETV4	ETS translocation variant 4	-3.48	*down-regulated*
RP11-514P8.6	Uncharacterized	0	*down-regulated*
IGSF1	Immunoglobulin superfamily member 1	-2.49	*down-regulated*
RASD1	Dexamethasone-induced Ras-related protein 1	-2.51	*down-regulated*
MRPL23	39S ribosomal protein L23mitochondrial	-3.47	*down-regulated*
CPNE4	Copine-4	-7.62	*down-regulated*
IL1RL1	Interleukin-1 receptor-like 1	-4.71	*down-regulated*
PI4KAP1	Pseudogene	-2.95	*down-regulated*
DLEC1	Deleted in lung and esophageal cancer protein 1	-3.45	*down-regulated*
ZBTB45P1	Pseudogene	0	*down-regulated*
LECT1	Leukocyte cell-derived chemotaxin 1	-2.67	*down-regulated*
SHC3	SHC-transforming protein 3	-3.28	*down-regulated*
SORCS1	VPS10 domain-containing receptor SorCS1	-2.95	*down-regulated*
STARD9	StAR-related lipid transfer protein 9	-2.34	*down-regulated*
TMSB4XP4	Pseudogene	-4.16	*down-regulated*
TMEM184A	Transmembrane protein 184^a^	-3.60	*down-regulated*
PCSK2	Neuroendocrine convertase 2	-2.22	*down-regulated*
FOSB	Protein fosB	-1.72	*down-regulated*
TRABD2A	Metalloprotease TIKI1	-3.42	*down-regulated*
GATM	Glycine amidinotransferase, mitochondrial	-1.82	*down-regulated*
BAG3	BAG family molecular chaperone regulator 3	-1.88	*down-regulated*
FAM105A	Inactive ubiquitin thioesterase FAM105A	-2.32	*down-regulated*
HSPA1B	Heat shock 70 kDa protein 1B	-2.42	*down-regulated*
MME	Neprilysin	-2.86	*down-regulated*
PDE4C	cAMP-specific 3’,5’-cyclic phosphodiesterase 4C	-3.47	*down-regulated*
RTN4RL2	Reticulon-4 receptor-like 2	-1.72	*down-regulated*
EDN3	Endothelin-3	-1.75	*down-regulated*
NRK	Nik-related protein kinase	-3.30	*down-regulated*
EGR2	E3 SUMO-protein ligase EGR2	-1.63	*down-regulated*
TNFRSF19	Tumor necrosis factor receptor superfamily member 19	-2.26	*down-regulated*
ABCC3	Canalicular multispecific organic anion transporter 2	-2.23	*down-regulated*
HSD17B6	17-beta-hydroxysteroid dehydrogenase type 6	-1.72	*down-regulated*
RP11-1212A22.1	Uncharacterized	-1.79	*down-regulated*
C15orf48	Normal mucosa of esophagus-specific gene 1 protein;NMES1	-2.57	*down-regulated*
GATA5	Transcription factor GATA-5	-4.48	*down-regulated*
FRAS1	Extracellular matrix protein FRAS1	-1.49	*down-regulated*
PCDH8	Protocadherin-8	-3.16	*down-regulated*
YJEFN3	YjeF N-terminal domain-containing protein 3	-2.36	*down-regulated*
SPHKAP	A-kinase anchor protein SPHKAP	-6.23	*down-regulated*
TBX2	T-box transcription factor TBX2	-1.60	*down-regulated*
KIAA1324	UPF0577 protein	-1.48	*down-regulated*
KIF5A	Kinesin heavy chain isoform 5A	-2.10	*down-regulated*
ARHGAP28	Rho GTPase-activating protein 28	-2.01	*down-regulated*
C1orf233	Fibronectin type-III domain-containing transmembrane protein	-1.94	*down-regulated*
NRG1	Pro-neuregulin-1, membrane-bound isoform	-1.83	*down-regulated*
HSPA1A	Heat shock 70 kDa protein 1A	-2.19	*down-regulated*
THBS1	Thrombospondin-1	-1.67	*down-regulated*
PLIN1	Perilipin-1	-2.51	*down-regulated*
XKRX	XK-related protein 2	-4.19	*down-regulated*

*FC: log2FoldChange.

**infinite: ratio between the number of sequences in normal tissue equal to 0 over the number of sequences found for multinodular goiter.

**Fig 2:**
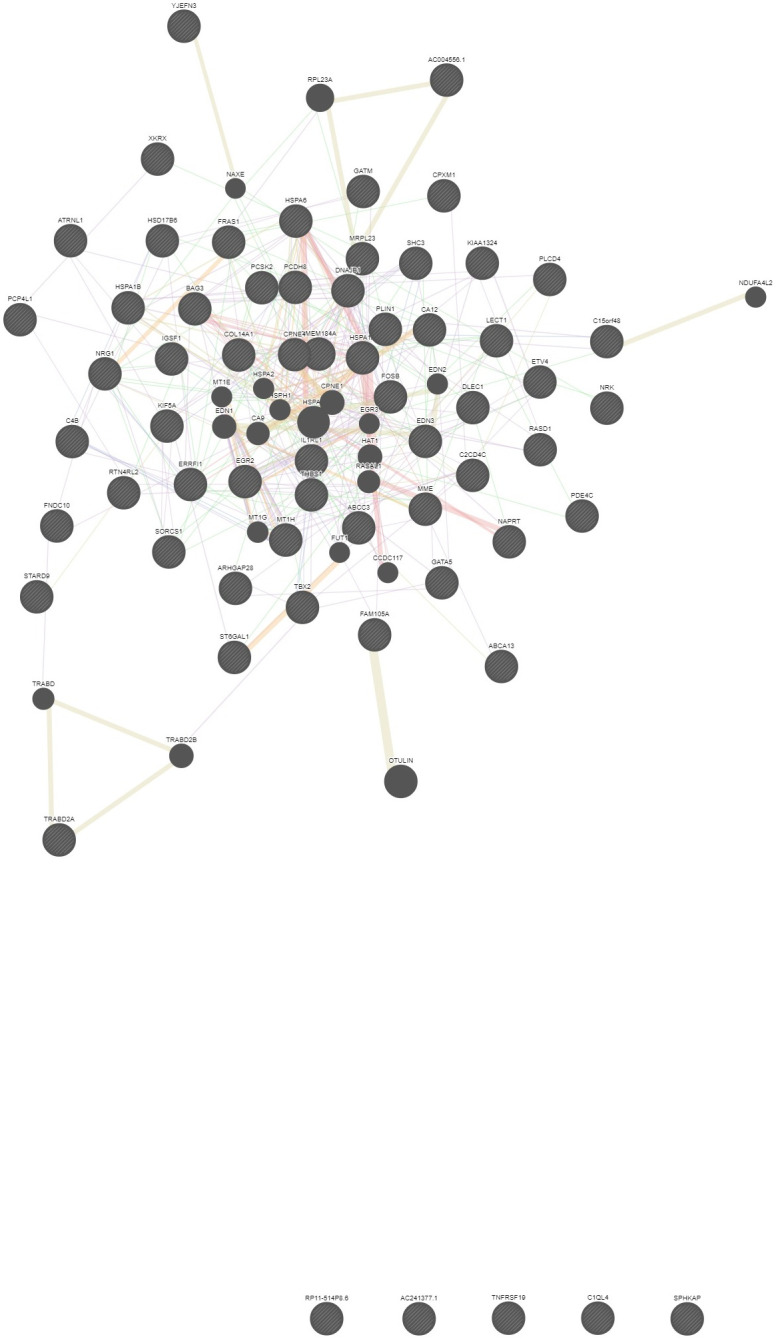
Protein-protein interaction analysis of 63 differentially expressed functional genes using the GeneMANIA software. A total of 423 gene interactions (more than 50% co-expression) of the five genes (TNF-RSF19, AC2413771 (HOTS gene), RP11-514P8.6, C1QL4, and SPHKAP) had no interactions identified with the others.

A biological interaction analysis between functional genes identified 423 possible interactions using the Gene MANIA tool with co-expression greater than 50% Fig 2.

## Discussion

Medical publications on thyroid surgical diseases are focused on the search for thyroid carcinoma biomarkers [23–26]. Initial immunohistochemistry and microarray studies comparing the expression profiles of normal, multinodular goiter, adenoma, and carcinoma tissue samples identified different patterns between the diseases but similarity between the groups of genes in the tissue with multinodular goiter and that with papillary carcinoma, which would explain the higher prevalence of incidental carcinoma (preoperatively unknown) in thyroids operated for multinodular goiter in goitrogenic areas and the existence of a common initial tumorigenesis factor [27, 28].

The literature is not clear about the molecular origin of multinodular goiter, which certainly involves epigenetic factors, heredity, and the classical iodine deficiency as well as iron and selenium deficiencies in the diet and exposure to foods rich in flavonoids and cyanogenic substances, such as cassava. When chronic, these conditions would lead to mutations and the onset of nodules in the gland [29, 30].

In medical practice, when facing thyroid nodules, the presence of malignant lesions needs to be considered [31, 32] along with the preoperative FNA investigation and Bethesda’s cytological classification, which often need to be repeated, present variable sensitivity and agreement with histopathology, and are inconclusive in up to 30% of cases [33, 34].

The need to identify which patients with thyroid nodule should undergo surgery, new therapy strategies, or clinical follow-up justifies the investigation of the molecular characteristics of lesions to determine the risk of multinodular goiter malignancy.

In this study, 70 sequences were differentially expressed between multinodular goiter and disease-free tissue. The down-regulated genes in multinodular goiter were related to several molecular pathways, especially phospholipase C (*PLCD4*), apoptosis pathways (*TNFRSF19*), heat shock proteins (*HSPA1A*, *HSPA6*), growth factors (*SHC3*, *NRG1*), *p53* proto-oncogene pathways (*THBS1*), and chaperone cell repair pathways (*BAG3*); on the other hand, the inflammatory (*COL14A)* and complement system (*C4B)* pathways were up-regulated in multinodular goiter tissue, in addition to the exclusive presence of an antisense transcription from the H19 locus, which encodes the nucleolar protein HOTS in multinodular goiter [35].

These findings are similar to the characteristics of tumor diseases with reduced apoptosis and cellular repair systems along with increased inflammatory activity in the presence of pro-tumor locus products, in this case the *HOTS* nucleolar protein, which, together with the lncRNA H19, would be possible inducers of cancerous breast, thyroid, liver, kidney, and lung lesions [36, 37].

The action of the *H19* gene and its products in tumor onset and hyperplastic lesions is evident, with the antagonism of *H19* lncRNA and *p53* and the activity of one of its gene products, *miR-675*, in promoting cellular and chromosomal instability being well described, as well as its hyperexpression in the presence of external factors such as hypoxia [37]. A balance between the products of sense (lncRNA) and antisense (*HOTS) H19* transcripts may be related to the regulation of cellular homeostasis.

There have been no studies on multinodular goiter using *NGS* and RNA-Seq with results similar to those described in the present study. In thyroid carcinoma, some lncRNA are discussed in the gene regulation of disease progression, such as *PTCSC3* [38], with *XLOC 051122* and *XLOC 006074* [39] in local metastasis and *PANDAR* as a possible target in pro-apoptotic therapies for carcinoma [40].

The question of whether the presence of multinodular goiter can be considered a risk factor for thyroid carcinoma still raises discussion. Recent findings have shown that the same histopathologically diagnosed papillary lesion exhibits different protein expression behavior if the patient has a history of multinodular goiter prior to the diagnosis of neoplasia [40–42].

Other studies have shown the importance of membrane proteins in the development of hyperplastic and neoplastic thyroid diseases, especially connexins and aquaporins [43–45]. This study identified no differences between the expression profiles of connexins or aquaporins in different tissues, but *STARD9* (apolipoprotein) and *CPNE4* membrane proteins were down-regulated in multinodular goiter.

Thus, it was possible to identify molecular characteristics of multinodular goiter similar to those found in the genesis of neoplastic tumor lesions, including: 1) reduced cell repair activity; 2) reduced apoptotic pathway activity; 3) increased inflammatory activity; and 4) *H19* gene expression with possible inhibitory activity of the *p53* proto-oncogene.

The presence of *H19* gene products hyper-expressed in multinodular goiter, a non-malignant disease with different forms of presentation in endemic regions (small and large multinodular goiters), contributes to the understanding of the genesis of multinodular goiter and its possible roles as a risk factor for malignant lesions and as a possible molecular marker.

Further studies in endemic areas with more replicates for *NGS* analysis and a better understanding of the function of ncRNAs in the development of the disease will be necessary to confirm the hypothesis of multinodular goiter as a pro-tumor state of the thyroid.

Previous findings in the literature have described the low expression of the *H19* gene and its products in thyroid cancer [46], which contradicts the high expression of this sequence in the multinodular goiter samples used in this study. This suggests that high *H19* gene expression may be used in conjunction with other molecular markers as a diagnostic tool in deciding between conservative and/or surgical treatment for multinodular goiter patients in endemic areas, such as the Amazon.

Future studies should further elucidate the molecular profile of multinodular goiter, deepen the understanding of the functions of non-coding RNA in malignant and non-malignant nodular diseases, and facilitate the development of rapid and cost-effective diagnostic protocols that consider the level of *H19* gene expression in patients with multinodular goiter.
